# How Public Health Professionals View Mandatory Vaccination in Italy—A Cross-Sectional Survey

**DOI:** 10.3390/vaccines9060580

**Published:** 2021-06-01

**Authors:** Erica Pitini, Valentina Baccolini, Annalisa Rosso, Azzurra Massimi, Corrado De Vito, Carolina Marzuillo, Paolo Villari

**Affiliations:** 1 Department of Public Health and Infectious Diseases, Sapienza University of Rome, P. le A. Moro 5, 00185 Rome, Italy; valentina.baccolini@uniroma1.it (V.B.); annalisa.rosso@uniroma1.it (A.R.); azzurra.massimi@uniroma1.it (A.M.); corrado.devito@uniroma1.it (C.D.V.); carolina.marzuillo@uniroma1.it (C.M.); paolo.villari@uniroma1.it (P.V.); 2 Local Health Unit-Azienda Sanitaria Locale Roma 1, Borgo Santo Spirito 3, 00193 Rome, Italy; 3 Local Health Unit-Azienda Sanitaria Locale Roma 2, V. le B. Bardanzellu 8, 00155 Rome, Italy

**Keywords:** mandatory vaccination, public health professionals, childhood vaccination, survey

## Abstract

In response to the decline in child vaccination coverage and the subsequent occurrence of large vaccine-preventable disease outbreaks, in 2017 Italy introduced a new law that made ten vaccines mandatory for children aged 0–16 years. The policy change initiated an ongoing debate among the general public, as well as in the political arena and the scientific community, over this major public health concern. Hence, we conducted a survey aimed at assessing Italian public health professionals’ attitudes towards and opinions on mandatory vaccination. A validated online questionnaire was administered to 1350 members of the Italian Society of Hygiene, Preventive Medicine and Public Health. Among the 1044 responders (response rate 77%), a large majority were in favour of the Italian mandatory vaccination law (91%) and against its repeal (74%). Nevertheless, according to our sample, maintaining a high level of vaccination coverage without the need to mandate would be preferable, and thus efforts to promote vaccine confidence and proactive vaccine uptake are still needed.

## 1. Introduction

In recent years, several developed countries have faced a decrease in childhood vaccine coverage and Italy has been no exception [1]. From 2013, national immunization coverage against poliomyelitis (by administration of a hexavalent vaccine that also protects against diphtheria, tetanus, pertussis, hepatitis B, and *Haemophilus influenzae* type b) began to fall below the 95% target [2]. National measles immunization coverage (by administration of the measles, mumps, and rubella (MMR) vaccine), already below the 95% threshold, started declining further after 2011, with the lowest coverage rate reported for 2015 (85.3%) [2,3]. This fall contributed to outbreaks in 2016—when 844 cases of measles were reported, compared with 251 in 2015—and in 2017, when 4991 measles cases were reported [4,5].

In response to this, in 2017 Italy introduced a new mandatory vaccination law (MVL) for children [6]. In fact, four vaccines were already mandatory and free of charge (those against diphtheria, tetanus, poliomyelitis, and hepatitis B), but there were no sanctions for non-compliers. The new law increased to ten the number of mandatory vaccines for children aged 0–16 years (adding those against pertussis, *Haemophilus influenza* type b, measles, mumps, rubella, and varicella), all free of charge. Moreover, it introduced sanctions for non-compliers, i.e., no admission into pre-school for unvaccinated children and financial penalties for parents of unvaccinated children attending compulsory schooling [6].

The scientific, political, and ethical debate raised by the new law is still open and involves not only citizens and politicians but also healthcare professionals [7,8]. Among the latter, public health professionals (PHPs) are heavily involved in vaccination programmes, but are also more generally concerned with driving policy interventions aimed at improving population health. On the matter of mandatory vaccination (MV), they must deal with various questions: “Is MV the best strategy for ensuring optimal vaccination coverage in Italy? Will it reduce vaccine-preventable disease morbidity? Is the application of the law sustainable from an organizational and economic point of view? Does the protection of public health take priority over individual rights?” Hence, we conducted a survey of a sample of Italian PHPs to assess their attitudes towards and opinions on MV.

## 2. Materials and Methods

### 2.1. Study Population

The survey addressed a random sample of 1350 PHPs from the Italian Society of Hygiene, Preventive Medicine and Public Health (Società Italiana di Igiene, Medicina Preventiva e Sanità Pubblica, SItI) [9]. The minimum sample size needed in the survey was estimated using the following inputs: population size, 2413 (all SIti members); proportion of the sample with the expected outcome (being in favour of the MV law), 70%; margin of error, ±3.0%; confidence level, 99.0%; and response rate, 70.0%.

### 2.2. Questionnaire

The questionnaire was developed on the basis of a narrative literature review and was piloted on a convenience sample of 73 SItI members [10]. It consisted of 45 questions grouped into five sections: I. Socio-demographic information (eight questions); II. Political and health system orientation (two questions); III. Personal and professional experience with vaccinations (four questions plus one conditional question); IV. Attitudes and opinions concerning MV (ten questions plus five conditional questions); V. Perception of the epidemiological, social, and economic impact of MV (fifteen questions) (see Supplementary Materials). Sections I to IV were assessed through closed-ended questions, some of which allowed multiple answers; section V was assessed through a five-point Likert scale. An invitation to participate in the survey was e-mailed to SItI members in June 2019. It included the details of the study, an internet link for the survey, and the statement that answering the questionnaire would constitute consent to participate in the study. To collect a large number of responses, and because we first invited the respondents at the beginning of the summer holidays, the survey availability was extended for five months. E-mail reminders were sent monthly. In an opinion dated 26 November 2018, the ethical committee of Sapienza University of Rome stated that the study did not require ethical approval as it used an anonymous questionnaire administered to experts.

### 2.3. Statistical Analysis

Statistical analysis was performed with Stata version 15.0 software (Stata Corporation, College Station, TX, USA). All data were processed anonymously. A descriptive analysis was performed using frequencies, percentages, mean values, standard deviations, and ranges. Multivariate logistic regression analysis was used to identify the determinants of attitudes towards MV and its impact. Regarding attitudes, the dependent variables “In favour of mandatory vaccination law”, “Mandatory vaccination law should be repealed”, and “In favour of flexible mandatory vaccination” were dichotomised by combining “No” and “Uncertain” as a negative response (Table S1a). Regarding the perception of the impact of MV, the dependent variables on the five-point Likert scale were dichotomised by collapsing ‘Strongly agree” and ‘‘Agree” into “Agree”, and ‘‘Disagree”, “Strongly disagree” and “Uncertain” into “Disagree” [11]; moreover, the answers to each sub-section (i.e., epidemiological, social, economic, and services impact) were combined and dichotomized into an overall outcome, i.e., “Optimistic” (if all the answers in the section showed a positive attitude) or “Not Optimistic” (if at least one answer in the section showed a negative attitude) (Table S1b). According to the Hosmer and Lemeshow logistic-regression model-building strategy, the variables examined by univariate analysis using the appropriate statistical test (chi-squared test or Fisher exact test for nominal variables and t-test for quantitative variables) were included in the model when the *p*-value was less than 0.25. Subsequently, final models were selected by backwards elimination of non-significant variables on the grounds of the likelihood-ratio test (cut-off *p*-value = 0.05) [12]. Results were expressed as adjusted odds ratios (ORs) and their 95% confidence intervals (CI) and *p*-values.

## 3. Results

### 3.1. Participation in the Survey and Characteristics of the Sample

Of the 1350 people invited to participate in the study, 1044 answered the questionnaire, giving a response rate of 77%. Respondents were predominantly of working age (mean 48.5 ± 14 years) and well distributed over the three areas of the country (Table 1). In terms of training, medical doctors prevailed (74%). Among those with a medical specialty, public health was the most frequent (86%) (Table S2). A large part of respondents (40%) worked in public health services, i.e., territorial services providing both population and individual interventions aimed at preventing disease and promoting health, such as health campaigns, health risk management, vaccinations, or behavioural counselling; one third worked in the academic sector, where research and educational activities prevail (Table 1).

Regarding experience with vaccinations, only one third of respondents were fully vaccinated or immunized as recommended for healthcare professionals (hepatitis B; influenza years 2018–2019; measles; mumps; rubella; varicella; pertussis) (Table S3b) [13]. In terms of knowledge, they felt well prepared on the subject of vaccinations (self-rated knowledge “good” or “excellent” for 78% of respondents) (Table S3a). About half of the respondents (54%) were directly involved with vaccinations in their professional activity. Finally, the years of professional experience with vaccinations for the overall sample ranged from 0 to 50 years (mean 7 years ± 11).

### 3.2. Attitudes towards Mandatory Vaccination

The vast majority of respondents (93%) embraced the general idea of MV for the protection of both individual and population health (Table 2 and Table S2). Moreover, respondents showed very positive attitudes towards the implementation of MV in Italy: 91% were in favour of the 2017 MVL and 74% were against its repeal. Notably, 78% did not support the most recent legislative proposal for a “flexible” MV, sponsored by the previous, populist government, where vaccinations would be made mandatory only when coverage rates fell appreciably. The main reason given for this aversion was that vaccinations are preventive measures that would lose effectiveness if used as reactive measures (Table S2). Regarding MMR vaccination, in order to achieve the global goal of eliminating endemic measles and rubella, 66% of respondents would make it mandatory for categories other than the 0–16 years age group, particularly healthcare professionals and school staff (Table 2).

#### Determinants of Attitudes towards the Mandatory Vaccination Law

Table 3 shows the results of the multivariate analysis. Being in favour of the MVL was positively associated with working in hospitals (OR 2.38; 95% CI 1.21–4.66) or the academic sector (OR 1.79; 95% CI 1.08–2.97), versus working in public health services, and being vaccinated or immunized as recommended (OR 2.47; 95% CI 1.41–4.27); a negative association was observed with support for a populist movement versus other political stances (OR 0.12; 95% CI 0.03–0.40). Supporters of a populist movement were also more likely to be in favour of repeal of the MVL (OR 3.35; 95% CI 1.11–10.1), as were respondents with more years of experience with vaccinations (OR 1.01; 95% CI 1.00–1.02). Finally, being in favour of flexible MV was positively associated with age (OR 1.02; 95% CI 1.00–1.03) but negatively associated with being vaccinated or immunized as recommended (OR 0.53; 95% CI 0.32–0.88).

### 3.3. Opinions on Mandatory Vaccination

All mandatory vaccines were considered of the utmost importance (very or extremely important) by the majority of respondents, with values ranging from 88% for *Haemophilus influenza* type b to 96% for measles, rubella, hepatitis B, and tetanus (Table 4a). Despite the positive attitudes towards MV, the majority of respondents (67%) selected “Promotion and information campaigns for the general population” as the best strategy for ensuring optimal vaccination coverage in Italy (Table 4b). Nevertheless, implementing vaccination strategies other than MV was considered quite difficult in the national context (very or extremely difficult for 74% of respondents), mainly due to organizational issues, as was ensuring optimal vaccination coverage in the absence of MV (very or extremely difficult for 87% of respondents).

### 3.4. Perception of the Impact of Mandatory Vaccination

This section was answered by 971 people. The answers revealed a positive perception of the epidemiological and economic impact of MV (Table 5 and Table S1b). On the epidemiological side, more than 90% of the sample agreed that MV increases vaccination coverage and reduces vaccine-preventable disease morbidity. On the economic side, despite uncertainty about the increase in costs for vaccination services, 87% of respondents agreed that, overall, MV will result in cost savings for the National Health Service (NHS). On the other hand, respondents were less optimistic about the social impact of MV and the impact on vaccination services (Table 5 and Table S1b). On the social side, respondents were divided over whether MV encourages hesitant parents to vaccinate their children (61%) and strengthens the anti-vaccine movement (54.5%). Surprisingly, given the positive attitudes towards the MVL, almost one third of the sample agreed that MV represents a failure of the Italian public health system (32%). By way of action, there was strong agreement that repealing the MVL would create confusion among citizens (87%). Perception of the impact of MV on vaccination services was unclear. In fact, although the majority of respondents disagreed with the statement that the organizational effort required for MV is unsustainable for vaccination services (62%), there was no common or clearly optimistic perception of the impact of MV on the workload of vaccination service staff, inconvenience for vaccination service users, and resource allocation in support of vaccination services.

#### Determinants of the Perception of the Impact of Mandatory Vaccination

In the multivariate analysis, the variable “Sector of work” was significantly associated with the perception of the impact of MV (Table 6). In particular, PHPs working in academia had a more optimistic perception of the epidemiological (OR 1.77; 95% CI 1.06–2.94), social (OR 1.58; 95% CI 1.01–2.47), and economic (OR 2.02; 95% CI 1.41–3.02) impact of MV, compared with those working in public health services; respondents working in hospitals were more optimistic about the economic (OR 2.06; 95% CI 1.47–2.78) and services impact (OR 1.95; 95% CI 1.03–3.70). Respondents who trusted the quality of the NHS were more optimistic about the social (OR 1.53; 95% CI 1.03–2.27) and services (OR 1.85; 95% CI 1.13–3.04) impact of MV. Public health professionals with more years of experience in the vaccination field were more optimistic about the economic impact of MV (OR 1.03; 95% CI 1.01–1.04), but less optimistic about the impact on vaccination services (OR 0.97; 95% CI 0.95–0.99). Older respondents were more optimistic about the social (OR 1.05; 95% CI 1.03–1.06) and services (OR 1.02; 95% CI 1.00–1.04) impact of MV. Finally, respondents with a non-medical degree were less likely to be optimistic about the epidemiological impact of MV (OR 0.56; 95% CI 0.36–0.89); supporters in the centre of the political spectrum were more likely to be optimistic about the social impact of MV (OR 2.60; 95% CI 1.27–5.31) than those on the left/centre left; and respondents working in the vaccination field were less likely to be optimistic about the economic impact of MV (OR 0.63; 95% CI 0.46–0.88).

## 4. Discussion

We explored the attitudes of Italian PHPs towards the current national MV policy, two years after its introduction, which provides ten mandatory vaccines for children aged 0–16 years. Overall, Italian PHPs were clearly in favour of the 2017 MVL. These data are consistent with a previous Italian study on healthcare workers (physicians, biologists, nurses, and midwives) where, out of 446 respondents, 78% expressed satisfaction with the 2017 MVL [14]. Moreover, a different study of 359 Italian public health residents showed that 69% agreed with the law and 88% deemed its mandatory nature necessary [15]. Outside the Italian context, relatively few studies have explored the attitudes of health care workers towards MV policies for the general population. In France, the percentage of GPs and paediatricians in favour of the previous MV policy there, which covered three compulsory vaccines (diphtheria, tetanus, and polio), was only 42% [16]. Nevertheless, no such data are available for the current French policy, introduced in 2018 in response to the fall in vaccination coverage, which made eight more vaccines compulsory for children up to two years of age (measles, mumps, rubella, pertussis, pneumococcus, hepatitis B, meningitis C, and *Haemophilus influenzae*) [17]. An increasing number of studies carried out in different countries have addressed the same topic from the perspective of the general population. A systematic review by Gualano et al. showed that, despite the growing resonance of anti-vaccination movements, most people seemed to be in favour of MV policies, with percentages ranging from 53% to 97% across Europe and North America [18].

Returning to PHPs, the most common reason given for supporting MV was the protection of the health of both the individual and the population. Notably, while the Italian MVL favours population safety over individual (or parental) wishes, it does not take priority over the right to education. In fact, school access for children of compulsory school age (6–16 years) whose parents refuse MV is still guaranteed, although the parents incur a financial penalty. The only exemptions from MV provided by the Italian law are for medical reasons. The situation is different in the US, where most states also offer some form of non-medical exemption from mandatory vaccination for school entry (i.e., religious exemptions in 45 states and Washington D.C.; philosophical exemptions in 15 states) [19]. Nevertheless, since non-medical exemptions could threaten community protection and result in an increased risk of disease outbreaks, numerous legislative proposals are calling for their complete removal or for additional requirements before an exemption can be granted [20].

Besides being in favour of MV, PHPs showed themselves to be very optimistic about the epidemiological and economic impacts of MV. Interestingly, respondents working in the academic sector, who are supposed to be more familiar with epidemiological and cost-effectiveness data, were significantly more optimistic about the epidemiological and economic impacts of MV than those working in public health services. On the epidemiological side, the vast majority of respondents were confident that the new law has increased vaccination coverage, in accordance with available data. Indeed, the national-level coverage rate for polio at 24 months of age increased from 93.3% in 2016 to 94.6% in 2017 (+1.3%) and reached the 95% coverage target in 2018 (+0.49%) and 2019 (−0.08%). The national-level coverage rate for measles at 24 months increased from 87.3% in 2016 to 91.8% in 2017 (+4.6%), 93.2% in 2018 (+1.38%), and 94.5% in 2019 (+1.27%), very close to the 95% coverage target [2]. This positive trend also included vaccine coverage for some non-mandatory vaccines (i.e., pneumococcal and meningococcal C). The international literature seems to confirm that MV policies are able to increase vaccine coverage [21,22]. However, in Italy, additional factors, such as the public debate and information campaigns that arose from the measles outbreaks and the introduction of the new law, may have increased awareness in the population of the importance of vaccination and thus the coverage level. Unfortunately, according to preliminary data for the year 2020, the COVID-19 pandemic is likely to have caused an alarming decline in vaccine uptake, as the fear of contagion and the adoption of containment measures by vaccine services has caused disruptions in both vaccine demand and supply [23]. On the economic side, most respondents believed that MV would prove to be cost-saving for the NHS. Although there are no data on the cost-effectiveness of mandatory vaccination programs to confirm this prediction, the scientific literature seems to recognize that, overall, vaccination programs are well worth the investment, not only because the healthcare costs of treating the prevented illnesses are averted, but also because of productivity gains driven by the improved performance of a healthier population [24,25].

Despite this enthusiasm, the results of the survey revealed some areas of criticism. First, while they endorsed the 2017 MVL, Italian PHPs believed that promotion and information campaigns aimed at the general population are the most desirable way to assure optimal vaccination coverage [26]. Nevertheless, carrying out vaccination strategies as an alternative to MV was considered very challenging, mainly due to organizational barriers, rather than lack of resources or political will [27]. In the end, MV appeared to be the easiest way to reach coverage targets in the national context, although not the most welcome. As a further point of interest, almost one third of the sample agreed that mandatory vaccination represents a failure of the Italian public health system.

Secondly, our results confirm that the introduction of MV in Italy has become a controversial political matter, mainly due to the strong opposition of populist forces, particularly the Five Star Movement, a non-conventional political party that eschews the traditional left–right paradigm. The survey results clearly show that PHPs who declared themselves supporters of a populist movement were significantly less favourable towards the MVL and were more in favour of its repeal than those with other political views were.

Other critical points arise from analysis of the perception of the social and services impact of MV, which, overall, was not particularly optimistic. Respondents working in public health services, and thus closer to the general public than other respondents, were significantly less optimistic about the social impact of MV than those working in academia. Regarding vaccine hesitancy and refusal, respondents were not sure whether MV would increase or decrease the confidence of citizens in vaccines; a small majority expected MV to counteract the hesitancy of parents to vaccinate their children, but at the same time to strengthen the anti-vaccine movement. A few surveys have explored the hesitancy and refusal of Italian parents to vaccinate their children. Unfortunately, the results of these surveys are not comparable over time because of heterogeneity in the target populations and survey methodologies [28,29,30,31,32]. Developing standardized instruments to monitor changes in parental attitudes towards vaccination over time would be a key step in assessing the social impact of MV. To comply with the requirements of the new law, local vaccination services are expected to deal with several challenges such as an increased number of appointments, intensified production of vaccination certificates, and identification of unvaccinated children in collaboration with educational services [8]. Our survey attempted to explore the impact of these requirements on vaccination services, but no clear picture emerged in terms of workload for vaccination service staff, resource allocation, or inconvenience to vaccination service users. On some level, this could be a good sign as no obvious signs of a breakdown in vaccination services were seen. Nevertheless, respondents working in public health services, and thus also in vaccination services, were less likely to feel optimistic about the impact of MV on such services, compared with those working in hospitals.

A final criticism is that only one third of the sample declared themselves to be fully vaccinated or immunized, as recommended for healthcare professionals by the Italian National Plan for Vaccine Prevention. Fully vaccinated PHPs were significantly more likely to favour the MVL and to dislike the proposal for the introduction of a flexible mandatory vaccination. Moreover, despite the near-unanimous agreement regarding MV for children, only 60% of respondents would make MMR vaccination mandatory for healthcare professionals, even though the transmission of the measles virus in healthcare settings remains a significant issue in Italy [33]. Although the context is different, the Covid-19 pandemic has renewed the debate around MV for healthcare workers, as vaccination plans worldwide require them to prioritize influenza and Covid-19 vaccines in order to protect themselves and their patients [34].

Beyond the above-mentioned criticisms, two policy indications seem to arise from the survey results. First, according to our sample of Italian PHPs, the 2017 MV law should not be repealed. There was strong agreement that removal of MV would create confusion among citizens. Secondly, the current MV law should not be revised in favour of a flexible approach, which would allow only temporary mandates in a regional and local epidemiological context. Such a proposal, which was put before Parliament by the 2018 populist government (comprising the Five Star Movement along with Lega, a far-right Party), has not been pursued further because of political instability at the time and, more recently, the Covid-19 pandemic.

While the use of a random sample should support the general applicability of the survey results to the entire population of Italian PHPs, our survey also has some limitations. The main one is that, since the Italian Society of Hygiene, Preventive Medicine and Public Health has long advocated for mandatory vaccination in Italy, a high share of acceptance of the 2017 MVL among its members was expected. Furthermore, although we promised absolute anonymity in the data analysis, and the questionnaire was self-administered, social desirability bias may still have decreased the reliability of some answers. Moreover, although the response rate was high, we were not able to compare the characteristics of respondents and non-respondents, as we had no data for non-respondents. In addition, collapsing the answers in each sub-section on the perception of the impact of MV into an overall outcome may have caused a loss of information, which could be remedied with further analysis. Finally, there remains the possibility that the experience of the Covid-19 pandemic may have changed the attitudes and opinions of PHPs and that a survey run during or after the pandemic may lead to different results.

## 5. Conclusions

Given the recent decrease in vaccination coverage and the subsequent outbreaks of vaccine-preventable diseases, Italian PHPs currently support the 2017 MV law and, in the short term, do not recommend its repeal or mitigation. Nevertheless, since maintaining high vaccination coverage without the need to mandate would be preferable in the long term, PHPs think that efforts must be made to promote vaccine confidence and proactive vaccine uptake, with a focus on health education and communication campaigns.

## Figures and Tables

**Table 1:** General characteristics of the survey sample (total = 1044).

Characteristic	Measure	
Gender, n, % of total		
Male	465	44.5
Female	579	55.5
Age (years), average (sd), range	48.5 (±14)	24–87
Region, n, % of total		
Northern Italy	405	38.8
Central Italy	261	25.0
Southern Italy and Islands	378	36.2
Academic degree, n, % of total		
Graduate	391	37.5
Post-graduate	653	62.5
Area of degree, n, % of total		
Medicine	776	74.3
Other health professions	128	12.3
Biology	107	10.2
Other	33	3.2
Sector of work, n, % of total		
Public health service	418	40.0
Academic	341	32.7
Hospital (public or private)	202	19.3
Government (national or local)/Technical agency	33	3.2
Other	50	4.8
Years of experience with public health, average, range	15.8 (±13)	0–61
Perceived quality of National Health Service, n, % of total		
Bad	3	0.3
Poor	97	9.3
Fair	311	29.8
Good	576	55.2
Excellent	57	5.4
Children aged 0–16, n, % of total		
No	778	74.5
Yes	266	25.5
Political stance, n, % of total		
Right/centre-right	125	12.0
Centre	51	4.9
Left/centre left	406	38.9
Populist movement *	13	1.2
Other	41	3.9
I do not want to answer	408	39.1

* In Italy, this entry refers to the “Five Star Movement”, a non-conventional political party that eschews the traditional left-right paradigm and is unanimously considered populist.

**Table 2:** Attitudes of public health professionals towards mandatory vaccination (total = 1044).

Attitudes	N (%)		
	*Yes*	*No*	*Uncertain*
In favour of MV	966 (92.5)	60 (5.8)	18 (1.7)
In favour of the 2017 Italian MV law	951 (91.0)	57 (5.5)	36 (3.5)
The 2017 MV law should be repealed	228 (21.8)	769 (73.7)	47 (4.5)
In favour of “flexible” MV	107 (10.2)	818 (78.4)	119 (11.4)
Would extend MMR MV to other groups	687 (65.8)	202 (19.4)	155 (14.8)
If yes, for which categories *			
Healthcare professionals	642 (93.5)		
School staff	577 (84.0)		
Soldiers	207 (30.1)		
Public administration	258 (37.6)		

* Multiple answers allowed. MV, mandatory vaccination; MMR, measles-mumps-rubella.

**Table 3:** Multivariate analysis: possible predictors of attitudes towards mandatory vaccination.

Model	OR	95% CI	*p*V
Model 1. In favour of MV law			
Sector of work			
Public health service (reference)	1.00		
Academic	1.79	1.08–2.97	0.024
Hospital	2.38	1.21–4.66	0.011
Government/Technical agency	0.95	0.32–2.86	0.938
Other	1.00		
Vaccinated or immunized as recommended *	2.47	1.41–4.27	0.001
Populist movement	0.12	0.03–0.40	0.001
Model 2. MV law should be removed			
Populist movement	3.35	1.11–10.1	0.032
Years of experience with vaccinations	1.01	1.00–1.02	0.016
Model 3. In favour of “flexible” MV			
Age	1.02	1.00–1.03	0.005
Vaccinated or immunized as recommended	0.53	0.32–0.88	0.015

* As recommended for healthcare professionals by the National Vaccine Prevention Plan 2017–2019. MV, mandatory vaccination; OR, odds ratio; *p*V, *p*-value.

**Table 4:** Opinions of public health professionals towards mandatory vaccination (total 1044).

**a. Importance of Mandatory Vaccines**					
**Mandatory Vaccine**			**N (%)**		
	**Not** **Important**	**Slightly** **Important**	**Moderately** **Important**	**Very** **Important**	**Extremely** **Important**
Poliomyelitis	2 (0.2)	11 (1.0)	69 (6.6)	250 (24.0)	712 (68.2)
Diphtheria	1 (0.1)	10 (0.9)	64 (6.1)	272 (26.1)	697 (66.8)
Anti-tetanus	2 (0.2)	4 (0.4)	32 (3.0)	247 (23.7)	759 (72.7)
Pertussis	1 (0.1)	9 (0.9)	54 (5.2)	314 (30.0)	666 (63.8)
Hepatitis B	1 (0.1)	3 (0.3)	38 (3.6)	245 (23.5)	757 (72.5)
*Haemophilus influenzae* b	2 (0.2)	17 (1.6)	110 (10.5)	340 (32.6)	575 (55.1)
Measles	1 (0.1)	3 (0.3)	38 (3.6)	222 (21.3)	780 (74.7)
Rubella	1 (0.1)	6 (0.6)	37 (3.5)	263 (25.2)	737 (70.6)
Mumps	1 (0.1)	8 (0.8)	76 (7.3)	335 (32.1)	624 (59.7)
Varicella	2 (0.2)	16 (1.5)	97 (9.3)	338 (32.4)	591 (56.6)
**b.** Alternative strategies to mandatory vaccinations					
					**N (%)**
The best strategies to ensure optimal vaccination coverage in Italy are *					
Mandatory vaccination					507 (48.6)
Promotion and information campaigns for the general population					695 (66.6)
Information and training campaigns for healthcare professionals					149 (14.3)
Organizational interventions aimed at strengthening vaccination services					304 (29.1)
Implementation of the national vaccination registry					242 (23.2)
Financial incentives for parents					16 (1.5)
Financial incentives for health professionals					10 (0.96)
It is difficult to implement alternative strategies to MV					
Not at all					1 (0.1)
Slightly					24 (2.3)
Moderately					249 (23.9)
Very					605 (57.9)
Extremely					165 (15.8)
Main barrier to the implementation of alternative strategies					
Lack of resources					243 (23.3)
Organizational issues					540 (51.7)
Lack of political will					159 (15.2)
Uncertain					30 (2.9)
Other					72 (6.9)
It is difficult to ensure vaccination coverage in the absence of MV					
Not at all					4 (0.4)
Slightly					49 (5.1)
Moderately					77 (7.9)
Very					466 (48.0)
Extremely					375 (38.6)

* At most two answers allowed. MV, mandatory vaccination.

**Table 5:** Perceived impact of mandatory vaccination among public health professionals (total 971).

Perceived Impact	N (%)				
	SD	D	NAD	A	SA
Epidemiological impact					
MV increases vaccination coverage for VPD	0 (0)	8 (0.8)	19 (2.0)	437 (45.0)	507 (52.2)
MV reduces VPD morbidity	4 (0.4)	28 (2.8)	57 (5.9)	489 (50.4)	393 (40.5)
Social impact					
MV increases citizens’ confidence in vaccines	44 (4.5)	333 (34.3)	290 (29.9)	252 (25.9)	52 (5.4)
MV encourages hesitant parents to vaccinate their children	20 (2.1)	159 (16.4)	203 (20.9)	472 (48.6)	117 (12.0)
MV reinforces anti-vaccine movements	30 (3.1)	203 (20.9)	209 (21.5)	403 (41.5)	126 (13.0)
MV damages relations between the State, health institutions, and citizens	140 (14.4)	463 (47.7)	213 (21.9)	136 (14.0)	19 (2.0)
MV represents a failure of Italian public health	164 (16.9)	363 (37.4)	130 (13.4)	230 (23.7)	84 (8.6)
MV removal would create confusion among citizens	19 (2.0)	42 (4.3)	70 (7.2)	410 (42.2)	430 (44.3)
Economic impact					
MV has significantly increased the costs for vaccination services	106 (10.9)	406 (41.8)	181 (18.7)	236 (24.3)	42 (4.3)
Overall, MV will result in cost savings for the National Health Service	10 (1.0)	25 (2.6)	89 (9.2)	463 (47.7)	384 (39.5)
Impact on vaccination services					
The organizational effort for MV is unsustainable for vaccination services	82 (8.4)	519 (53.5)	196 (20.2)	133 (13.7)	41 (4.2)
MV has resulted in an excessive workload for vaccination service staff	60 (6.2)	354 (36.5)	161 (16.6)	294 (30.2)	102 (10.5)
MV has caused inconvenience to vaccination service users	41 (4.2)	297 (30.6)	237 (24.4)	333 (34.3)	63 (6.5)
MV was sustained by adequate resources for vaccination services	136 (14.0)	331 (34.1)	297 (30.6)	190(19.6)	17 (1.7)
MV has diverted resources away from other vaccination activities	107 (11.0)	390 (40.2)	299 (30.8)	134(13.8)	41 (4.2)

SD, strongly disagree; D, disagree; NAD, neither agree nor disagree; A, agree; SA, strongly agree; MV, mandatory vaccination; VPD, vaccine preventable disease.

**Table 6:** Multivariate analysis: possible predictors of the perception of the impact of MV.

Model	OR	95% CI	*p*V
Model 4. Optimistic Regarding the Epidemiological Impact of MV			
Area of degree other than medicine	0.56	0.36–0.89	0.013
Sector of work			
Public health service (reference)	1.00		
Academic	1.77	1.06–2.94	0.028
Hospital	1.51	0.82–2.78	0.188
Government/Technical agency	0.89	0.29–2.65	0.829
Other	3.19	0.75–13.6	0.117
Model 5. Optimistic regarding the social impact of MV			
Age	1.05	1.03–1.06	<0.001
Sector of work			
Public health service (reference)	1		
Academic	1.58	1.01–2.47	0.043
Hospital	1.44	0.83–2.51	0.197
Government /Technical agency	0.98	0.31–3.04	0.967
Other	2.59	1.25–5.36	0.010
Political stance			
Left/centre-left (reference)	1.00		
Right/centre-right	1.70	0.95–3.04	0.075
Centre	2.60	1.27–5.31	0.009
Populist movement	2.25	0.42–12.1	0.347
Other	0.83	0.29–2.31	0.716
I do not want to answer	1.07	0.68–1.67	0.771
Perceived quality of NHS more than fair	1.53	1.03–2.27	0.037
Model 6. Optimistic regarding the economic impact of MV			
Sector of work			
Public health service (reference)	1		
Academic	2.02	1.41–3.02	<0.001
Hospital	2.06	1.47–2.78	<0.001
Government /Technical agency	1.00	0.47–2.12	0.996
Other	3.01	1.50–6.09	0.002
Professional activity dealing with vaccinations	0.63	0.46–0.88	0.006
Years of experience with vaccinations	1.03	1.01–1.04	0.001
Model 7. Optimistic regarding the impact of MV on vaccination services			
Age	1.02	1.00–1.04	0.006
Sector of work			
Public health service (reference)	1.00		
Academic	1.63	0.93–2.86	0.090
Hospital	1.95	1.03–3.70	0.040
Government /Technical agency	2.13	0.68–6.66	0.195
Other	3.80	1.67–8.64	0.001
Perceived quality of NHS more than fair	1.85	1.13–3.04	0.014
Years of experience with vaccinations	0.97	0.95–0.99	0.037

MV, mandatory vaccination; OR, odds ratio; *p*V, *p*-value.

## Data Availability

The data presented in this study are available on request from the corresponding author.

## Supplementary Materials

The following are available online at https://www.mdpi.com/article/10.3390/vaccines9060580/s1: Survey questionnaire; Table S1: Dependent variables dichotomization for univariate and multivariate analysis; Table S2: Answers to the conditional questions of the survey; Table S3: Professional and personal experience with vaccination.
