# Supplementary material for: How Public Health Professionals View Mandatory Vaccination in Italy—A Cross-Sectional Survey
Source: Vaccines (Basel). 2021 Jun 1;9(6):580. doi: 10.3390/vaccines9060580 (PMC8228801; doi:10.3390/vaccines9060580)
Supplement: Supplementary file 1 [file vaccines-09-00580-s001.zip › Pitini_suppl_1_07_05.pdf]

SUPPLEMENT 1

'How public health professionals view mandatory vaccination in Italy – A cross-sectional survey'

SECTION I – SOCIO-DEMOGRAPHIC INFORMATION

1.1 Gender:

Male
Female

1.2 Birth year: ____________________

1.3 In which Province do you conduct your professional activity? ____________________

1.4 What is the highest educational degree obtained?

Triennal degree
I level Master's
Master's degree
II level Master's
Medical specialty/ PhD

1.5 Area of degree:

Medicine, specify medical specialty if achieved ____________________
Health professions, specify ____________________
Biology
Other, specify ____________________

1.6 What sector do you work in?

Public hospital
Private hospital
Prevention department
District
Other territorial structure
Accademy
National Government (e.g., Ministry of Health)
Local Government (e.g., Regional Agency)
Technical/Research agency (e.g., Istituto Superiore di Sanità, Agenas)
Other, specify ____________________

1.7 Years of professional experience in Hygiene and Preventive Medicine: ____________________

1.8 Do you have children aged 0-16?

Yes
No

SECTION II – POLITICAL AND HEALTH SYSTEM ORIENTATION

2.1 How would you define your political stance?

Right
Center-right
Center
Center-left
Left
Populist movements
Other, specify ____________________
Do not want to answer

2.2 How would you rate the quality of the Italian National Health System?

Poor
Inadequate
Adequate
Good
Excellent

SECTION III – PERSONAL AND PROFESSIONAL EXPERIENCE WITH VACCINATIONS

3.1 Are you vaccinated or immunized against the following infectious diseases, as recommended for healthcare professionals by the National Vaccine Prevention Plan 2017-2019?

	Yes	No	Do not remember
**Hepatitis B**			
**Influenza (year 2018-19)**			
**Measles**			
**Mumps**			
**Rubella**			
**Varicella**			
**Pertussis**			

3.2 How would you rate your knowledge about vaccinations?

Poor
Inadequate
Adequate
Good
Excellent

3.3 Does your professional activity deal with vaccinations?

Yes
No

3.3.a If yes, does it deal particularly with:

Delivery of vaccines

Organization of vaccination services
Research
Education
Other, specify ____________________

3.4 Years of professional experience in the field of vaccinations___________________

SECTION IV – ATTITUDES AND OPINIONS ON MANDATORY VACCINATION

ATTITUDES

4.1 In general, are you in favor of mandatory vaccination?

Yes
No
I do not know

4.1.a If yes, why?

It protects the health of individuals and population
It is a good strategy for achieving the European goals for immunization and control of vaccine-preventable diseases
It is a good strategy for overcoming vaccine hesitancy
It ensures equity (equal distribution of risks and benefits in the population)
Others, specify ____________________

4.1.b If no, why?

It is a coercive measure that harms people's freedom and rights
It is a simplistic solution that does not take into account the complexity of the problem
It strengthens anti-vaccine movements
It is a paternalistic approach that disempowers people about their health
Other, specify____________________

4.2 Are you in favour of the 2017 Italian mandatory vaccination law?

Yes
No
I do not know

4.3 Do you think that the 2017 mandatory vaccination law should be repealed?

Yes, immediately
Yes, gradually
No
I do not know

4.4 Are you in favour of the legislative proposal for a "flexible" mandatory vaccination sponsored by the recent populist government, where vaccinations are made mandatory only when the rate of vaccination coverage falls markedly?

Yes

No
I do not know

4.4 a If yes, why?

It represents the right balance between the protection of personal freedom and the protection of public health
It limits the number of mandatory vaccines to those that are strictly necessary
It is a good strategy for improving relationships with anti-vaccine movements
Other, specify ____________________

4.4 b If No, why?

Vaccinations are preventive measures that would lose effectiveness if used as reactive measures
It would be very difficult to ensure adherence to mandatory vaccination in emergency situations
There is still no information system capable of guaranteeing accurate monitoring of vaccination coverage in all Italian Regions (regional and national vaccination registries)
At present, application of the "flexible" option would still be necessary, so it is worth keeping the current obligation in place
Other, specify ____________________

4.5 Would you make MMR vaccination mandatory in categories other than the 0-16 age group, in order to achieve the goal of eliminating endemic measles and rubella?

Yes
No
I do not know

4.5 a If yes, for which of the following categories (multiple answers allowed)?

Healthcare professionals
School staff
Military personnel
Public administration
Other, specify ____________________

OPINIONS

4.6 How would you rate the importance of the following mandatory vaccinations?

	Not important	Weakly important	Moderately important	Very important	Extremely important
**Poliomyelitis**					
**Diphtheria**					
**Anti-tetanus**					
**Pertussis**					
**Hepatitis B**					
***Haemophilus influenzae* b**					
**Measles**					
**Rubella**					
**Mumps**					
**Varicella**					

4.7 What are the best strategies for ensuring optimal vaccination coverage in Italy (multiple answers allowed)?

Mandatory vaccination
Promotion and information campaigns for the general population
Information and education campaign for healthcare workers
Organizational interventions aimed at strengthening vaccination services
Implementation of the national vaccination registry
Financial incentives for parents
Financial incentives for healthcare professionals
Other, specify ____________________

4.8 How hard is it to implement alternative strategies to mandatory vaccination in Italy?

Extremely
Very
Moderately
Slightly
Not at all

4.9 How hard is it to ensure vaccination coverage in the absence of mandatory vaccination in Italy?

Extremely
Very
Moderately
Slightly
Not at all

4 .10 What is the main barrier to the implementation of alternative strategies to mandatory vaccination in Italy?

Lack of resources
Organizational issues
Lack of political will
Uncertain
Other, specify____________________

SECTION V – PERCEPTION OF THE IMPACT OF MANDATORY VACCINATION

5.1 How far do you agree with the following statements

EPIDEMIOLOGICAL IMPACT

	Strongly disagree	Disagree	Neither agree nor disagree	Agree	Strongly agree
**5.1a** Mandatory vaccination increases vaccination coverage for VPD					
**5.1b** Mandatory vaccination reduces VPD morbidity					

SOCIAL IMPACT

	Strongly disagree	Disagree	Neither agree nor disagree	Agree	Strongly agree
**5.1c** Mandatory vaccination increases citizens' confidence in vaccines					
**5.1d** Mandatory vaccination encourages hesitant parents to vaccinate their children					
**5.1e** Mandatory vaccination strengthens anti-vaccine movements					
**5.1f** Mandatory vaccination damages relations between the State, health institutions and citizens					
**5.1g** Mandatory vaccination represents a failure of the Italian public health system					
**5.1h** Mandatory vaccination repeal would create confusion among citizens					

ECONOMIC IMPACT

	Strongly disagree	Disagree	Neither agree nor disagree	Agree	Strongly agree
**5.1i** Mandatory vaccination has significantly increased the costs of vaccination services					
**5.1l** Overall, mandatory vaccination will result in cost savings for the National Health Service					

IMPACT ON VACCINATION SERVICES

	Strongly disagree	Disagree	Neither agree nor disagree	Agree	Strongy agree
**5.1m** The organizational effort for mandatory vaccination is unsustainable for vaccination services					
**5.1n** Mandatory vaccination has resulted in an excessive workload for vaccination service staff					
**5.1o** Mandatory vaccination has caused inconvenience to vaccination service users					
**5.1p** Mandatory vaccination was sustained by an increase in resources devoted to vaccination services					
**5.1q** Mandatory vaccination has diverted resources away from other vaccination-related activities					

VPD, vaccine-preventable diseases
