# Supplementary material for: How Public Health Professionals View Mandatory Vaccination in Italy—A Cross-Sectional Survey
Source: Vaccines (Basel). 2021 Jun 1;9(6):580. doi: 10.3390/vaccines9060580 (PMC8228801; doi:10.3390/vaccines9060580)
Supplement: Supplementary file 1 [file vaccines-09-00580-s001.zip › Pitini_suppl_2_04_05.pdf]

Table S1 Dependent variables dichotomization for univariate and multivariate analysis

a. Attitudes towards mandatory vaccination (total 1044)

	N (%)	
	Yes	No
In favour of the 2017 Italian MV law	951 (91.1)	93 (8.9)
The 2017 MV law should be repealed	228 (21.8)	816 (78.2)
In favour of "flexible" MV	107 (10.2)	937 (89.8)

MV, mandatory vaccination

b. Perception of the impact of mandatory vaccination (total 971)

	N (%)	
	Yes	No
Optimistic regarding the epidemiological impact of MV	875 (90.1)	96 (9.8)
Optimistic regarding the social impact of MV	141 (14.5)	830 (85.5)
Optimistic regarding the economic impact of MV	606 (62.4)	365 (37.6)
Optimistic regarding the impact of MV on vaccination services	89 (9.2)	882 (90.8)

MV, mandatory vaccination

Table S2 Answers to the conditional questions of the survey.

Question	N	%
Medical specialty if achieved [535 observations]		
Public Health	458	85.6
Microbiology/infectious diseases	15	2.8
Pediatrics	11	2.0
Occupational medicine	18	3.4
Other	33	6.2
Professional activity dealing with vaccinations: specific field of activity [567 observations]		
Delivery of vaccines	208	36.7
Organization of vaccination services	147	25.9
Research	101	17.8
Education	85	15.0
Other	26	4.6
In favor of MV: reason [966 observations]		
It protects the health of individuals and population	649	67.2
It is a good strategy for achieving the European goals for control VPD	207	21.4
It is a good strategy for overcoming vaccine hesitancy	56	5.8
It ensures equity (equal distribution of risks and benefits in the population)	38	3.9
Other	16	1.7
Not in favor of MV: reason [60 observations]		
It is a coercive measure that harms people's freedom and rights	1	1.7
It is a simplistic solution that does not take into account the problem complexity	23	38.3
It strengthens anti-vaccine movements	2	3.3
It is a paternalistic approach that disempowers people about their health	32	53.4
Other	2	3.3
In favor of flexible MV: reason [107 observations]		
It is the right balance between the protection of personal freedom and public health	84	78.5
It limits the number of mandatory vaccines to those that are strictly necessary	11	10.3
It is a good strategy for improving relationships with anti-vaccine movements	7	6.5
Other	5	4.7
Not in favor of flexible MV: reason [818 observations]		
Vaccinations are preventive measures not effective as reactive measures	547	66.9
It would be very difficult to ensure adherence to MV in emergency situations	83	10.1
There is no information system for the monitoring of vaccination coverage	114	13.9
At present, application of the "flexible" option would still be necessary	40	4.9
Other	34	4.2
Would extend MPR MV to other groups		

Only one answer allowed for each question. VPD, vaccine-preventable diseases; MV, mandatory vaccinations

Table S3 Professional and personal experience with vaccination (total 1044):

a. Professional experience;

Experience	Measure	
Professional activity dealing with vaccinations, n, % of total		
No	477	45.7
Yes	567	54.3
Years of experience with vaccinations, average, range	7.1(±11)	0-50
Self-rated knowledge about vaccinations, n, % of total		
Bad	2	0.2
Poor	30	2.9
Fair	194	18.6
Good	545	52.2
Excellent	273	26.1

b. Personal experience

Vaccinated or immunized against *	N (%)		
	Yes	*No*	*DNR*
Hepatitis B	895 (85.7)	130 (12.5)	19 (1.8)
Influenza (year 2018-19)	745 (71.4)	298 (28.5)	1 (0.1)
Measles	740 (70.9)	257 (24.6)	47 (4.5)
Mumps	697 (66.8)	291 (27.9)	56 (5.3)
Rubella	715 (68.5)	268 (25.7)	61 (5.8)
Varicella	637 (61.0)	374 (35.8)	33 (3.2)
Pertussis	668 (64.0)	304 (29.1)	72 (6.9)
All of the above	344 (33.0)	700 (67.0)	-

*As recommended for healthcare professionals by the National Vaccine Prevention Plan 2017-2019.
DNR, do not remember.
